# Exploring the mediating role of serum vitamin D in the link between dietary live microbes intake and obesity: a cross-sectional real-world study

**DOI:** 10.3389/fnut.2025.1588700

**Published:** 2025-08-29

**Authors:** Chan Liu, Huiling Zheng, Wenming Xu, Xiaoxiao Zhu, Mei Li, Lijuan Liu

**Affiliations:** ^1^Key Laboratory of Nephrology, Guangdong Provincial Key Laboratory of Nephrology, Department of Nephrology, Ministry of Health of China, The First Affiliated Hospital of Sun Yat-sen University, Guangzhou, China; ^2^Department of Health Management Centre, The First Affiliated Hospital of Sun Yat-sen University, Guangzhou, China; ^3^Department of Endocrinology, The First Affiliated Hospital of Sun Yat-sen University, Guangzhou, China; ^4^Department of General Practice, The First Affiliated Hospital of Sun Yat-sen University, Guangzhou, China; ^5^Department of Gastroenterology, The First Affiliated Hospital of Sun Yat-sen University, Guangzhou, China

**Keywords:** live microbes intake, NHANES, vitamin D, obesity, mediation analysis

## Abstract

**Background:**

Existing research results suggest a correlation between body mass index (BMI) and serum vitamin D levels, as well as the intake of live microbes from dietary sources. However, it is essential to further investigate whether serum vitamin D could serve as a mediator in the relationship between the consumption of dietary live microbes and obesity, as this connection remains to be elucidated.

**Methods:**

We analyzed data from 18,099 participants in the 2007–2018 National Health and Nutrition Examination Survey (NHANES), focusing on obesity [assessed via BMI and waist circumference (WC)], serum vitamin D levels, and the dietary intake of live microbes (evaluated both as a continuous variable and a three-level categorical variable). A composite category “MedHi” was used to reflect the intake of foods containing medium (10^4^–10^7^ colony-forming units (CFU/g)) or high (>10^7^ CFU/g) levels of live microbes. Mediation analysis was conducted to explore how serum vitamin D potentially mediates the relationship between the dietary intake of live microbes and obesity.

**Results:**

After adjusting for potential confounding factors, it was found that both vitamin D and the MedHi consumption were strongly and negatively associated with obesity. Mediation analysis revealed that serum vitamin D mediated the relationship between the dietary intake of live microbes and BMI, WC, obesity, and abdominal obesity with mediated proportions of 14.6, 12.5, 13.0, and 12.5%, respectively.

**Conclusion:**

The positive association between the dietary intake of live microbes and obesity risk is partly mediated by serum vitamin D. Foods with higher microbial concentrations could be beneficial.

## 1 Introduction

Obesity, traditionally defined as a body mass index (BMI) ≥30 kg/m^2^, has experienced a steady annual increase in recent years (1). This condition is crucially linked to a higher incidence of cardiovascular, digestive, respiratory, nervous, musculoskeletal, and infectious diseases (2). Moreover, compared with individuals with a normal weight, those with obesity are at a significantly greater risk of developing multiple diseases. In summary, the escalating prevalence of obesity and the consequent increase in disease burden pose critical challenges that urgently require attention.

Generally, overweight and obese individuals have three clinical weight loss options: (a) lifestyle changes (diet, exercise, and behavioral therapy), (b) pharmacotherapy, and (c) bariatric surgery (3). Previous research has shown that incorporating probiotics can help reduce the obesity index (4). Interestingly, an association has been observed between the consumption of foods rich in live microbes and lower measures of BMI and WC (5). Additionally, observational studies have linked low vitamin D levels with obesity (6, 7). Experimental research has further demonstrated that low vitamin D may contribute to obesity by affecting adipose tissue differentiation and growth through the regulation of gene expression or the modulation of parathyroid hormone, calcium, and leptin levels (8). Some findings suggest a relationship between vitamin D status and the composition and diversity of the gut microbiota (9).

Vitamin D, a pleiotropic steroid hormone, plays a crucial role in regulating calcium homeostasis and bone mineralization. Recent research has broadened our understanding of its functions, linking vitamin D to various conditions such as obesity, diabetes (DM), cardiovascular diseases (CVD), immune regulation, and cancer (10–12). While there is no consensus on the optimal levels of 25-hydroxyvitamin D in serum, most experts define vitamin D deficiency as a level below 20 ng/mL (50 nmol/L) (13). Causes of vitamin D deficiency include reduced skin synthesis, impaired absorption, and both acquired and heritable disorders affecting vitamin D metabolism and responsiveness (14).

**Figure 1:**
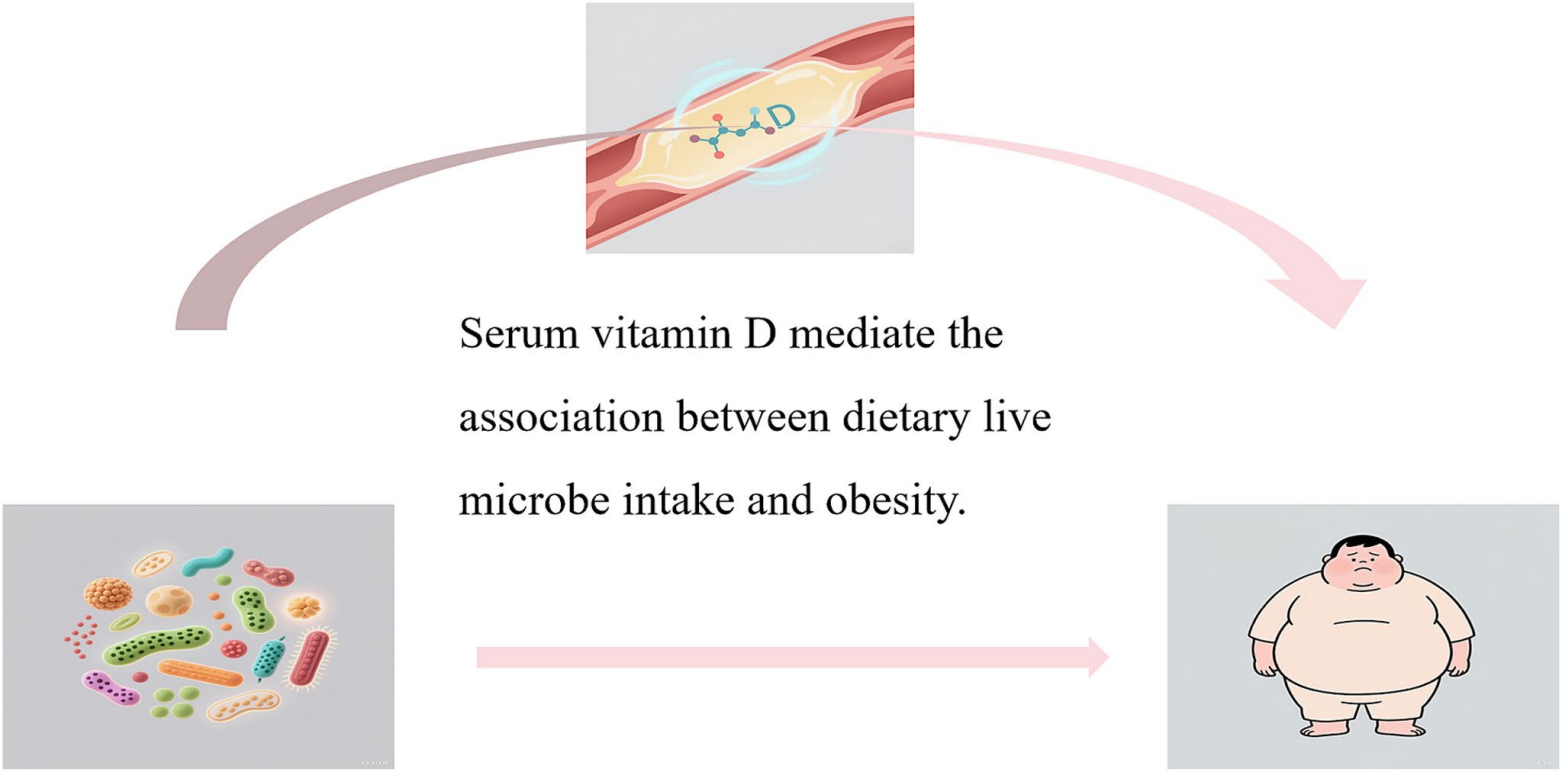
Hypothetical conceptual framework diagram.

To investigate the potential relationships among dietary intake of live microbes, serum vitamin D levels, and obesity, we conducted a comprehensive analysis using data from a nationally representative sample of the U.S. population. Multiple statistical approaches were employed, including regression and mediation analyses, to assess both direct and indirect associations. Specifically, we explored whether serum vitamin D levels could serve as a mediating factor in the relationship between live microbe intake and obesity outcomes. A conceptual framework illustrating this hypothesized mediation pathway is presented in Figure 1.

## 2 Methods

### 2.1 Study design and participants

The study utilized participants from the National Health and Nutrition Examination Survey (NHANES) program,^1^ a series of biennial cross-sectional health surveys conducted among the general U.S. population since 1999. We selected NHANES because it provides nationally representative, population-based data on dietary intake, biochemical indicators, and anthropometric measures, with rigorous quality control and standardized data collection protocols. This makes NHANES highly suitable for examining the complex relationships between nutrition, biomarkers (such as vitamin D), and obesity outcomes in a real-world context. These surveys gather comprehensive data on nutritional intake, health behaviors, and medical conditions. Trained interviewers performed in-home interviews, collecting detailed demographic, dietary, socioeconomic, and health-related information using standardized questionnaires (15). Physical examinations were conducted at a mobile examination center, as described in the NHANES documentation. We included data from six cycles spanning from 2007 to 2018. We chose this period because it ensured consistent measurement of serum vitamin D using liquid chromatography–tandem mass spectrometry (LC-MS/MS) and provided comprehensive and comparable data on dietary intake, anthropometry, and relevant covariates. All participants provided written informed consent, and the National Center for Health Statistics (NCHS) Research Ethics Review Board approved the survey protocol. As this study constituted a secondary analysis of deidentified data, it did not require additional institutional review board approval.

### 2.2 Study population

**Figure 2:**
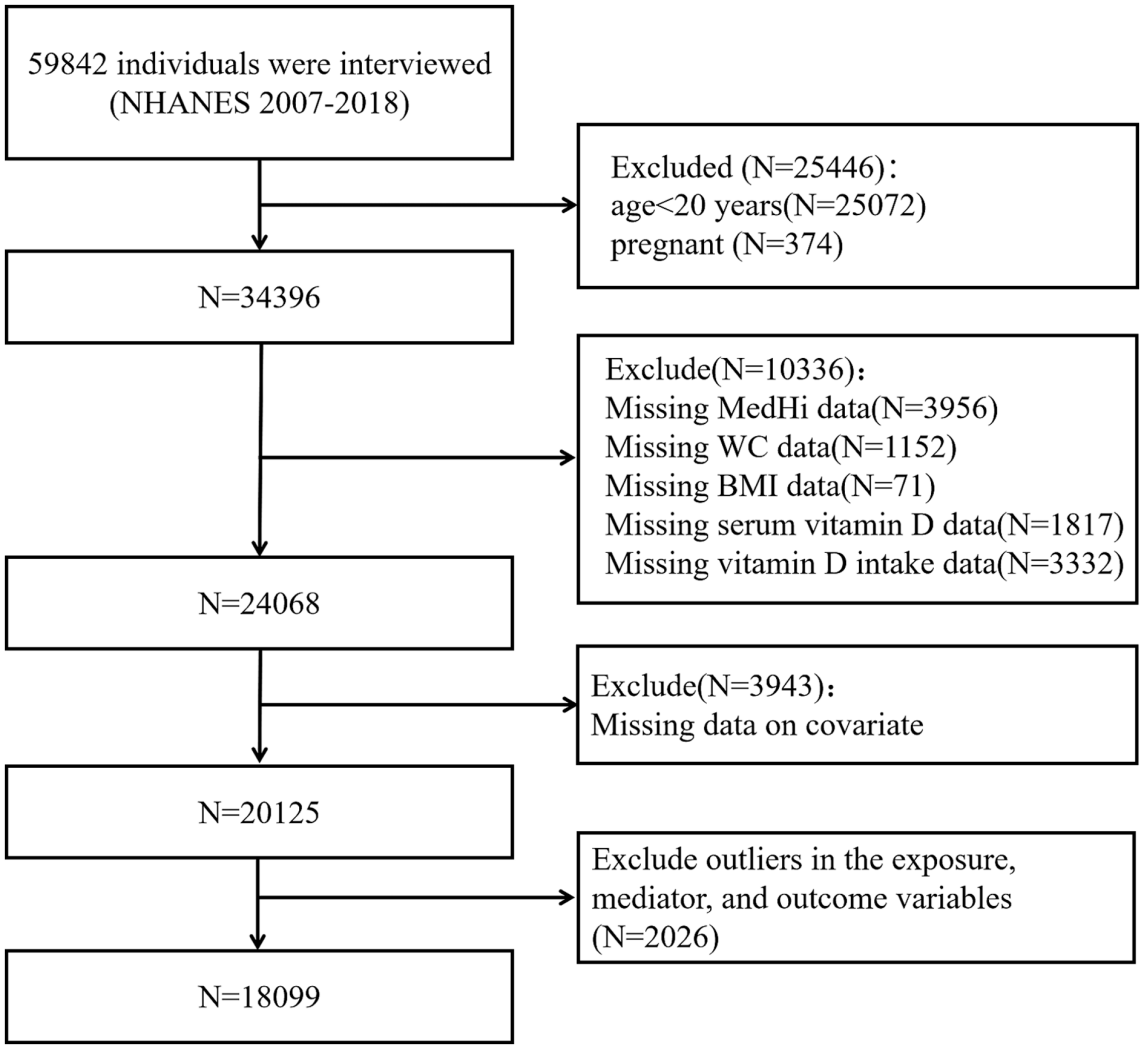
Study flowchart of the participants.

We restricted our study sample to adults aged ≥20 years to ensure comparability with previous research on obesity and vitamin D status in adult populations. Pregnant participants were excluded due to altered vitamin D metabolism and body composition during pregnancy, which could confound the analysis. Specifically, individuals with outlier values in BMI and WC were identified through distributional diagnostics and excluded from the final analysis. Participants with missing data on key exposure, mediator, or outcome variables were excluded to maintain the integrity of the mediation analysis. Participants with extreme outlier values in the exposure (MedHi), mediator (serum vitamin D), and outcome variables (BMI and WC) were excluded based on visual inspection of their distributions and standardized thresholds. Our final analytical sample comprised 18,099 participants. The inclusion and exclusion criteria are summarized in Figure 2.

### 2.3 Dietary intakes and live microbial category

Dietary intake data were obtained by comparing the 24-h dietary recall with the USDA Food and Nutrient Database. Marco et al. (16) developed a method to estimate live microbial content in 9,388 food codes from NHANES, classifying foods into three categories: low (<10^4^ CFU/g), medium (10^4^–10^7^ CFU/g), and high (>10^7^ CFU/g) microbial levels. According to the classification system developed by Marco et al. (16), fermented dairy products comprised the majority of foods assigned to the high microbial (Hi) category. Specifically, yogurts and other cultured milks were typically classified as Hi unless consumed as a minor ingredient in composite dishes. Most cheeses, including fresh and soft varieties, were also assigned to the Hi category, with exceptions for long-aged cheeses (e.g., Parmesan), processed cheeses (e.g., American cheese), and cheeses in cooked items such as pizza. Cheese-containing sandwiches were classified as Hi only if they contained non-processed cheese and were not heated before consumption. These classification criteria were used to estimate the microbial exposure for each food item in NHANES dietary recalls.

A composite category, “MedHi,” was created to represent individuals consuming foods from either the medium or high microbial content groups. This classification was based on a literature review and expert consensus, considering microbial viability after food processing.

Participants were then grouped using two approaches. First, based on MedHi intake, they were categorized into G1 (no MedHi intake), G2 (intake >0 but < median), and G3 (intake ≥ median). Second, based on overall dietary microbial exposure, participants were grouped as low (only Lo-category foods), moderate (Med-category but no Hi-category foods), or high (any Hi-category foods). These groupings were used in both continuous and categorical analyses.

### 2.4 Obesity

Obesity was defined as BMI ≥30 kg/m^2^ and abdominal obesity as WC >102 cm in men and >88 cm in women based on World Health Organization (WHO) criteria (17). BMI was calculated as weight (kg) divided by height (m^2^).

Anthropometric data, such as height, weight, and WC, were obtained by trained technicians following standardized NHANES protocols. Although these measurements are considered highly reliable, we did not apply formal corrections for measurement error. All analyses assume non-differential error across exposure groups.

### 2.5 Measurement of vitamin D serum levels

The total serum vitamin D levels were measured using ultra-high-performance liquid chromatography–tandem mass spectrometry. The analytes were separated using either a pentafluorophenyl (PFP) column (Hypersil GOLD PFP, 2.1 mm × 100 mm, 1.9 μm) from Thermo Scientific (Waltham, MA, United States) or a Kinetex PFP column (2.1 mm × 100 mm, 1.7 μm) from Phenomenex (Torrance, CA, United States). For a comprehensive description of the methodology, please refer to the CDC’s analytical notes available at www.cdc.gov/nchs/nhanes/vitamind/analyticalnote.aspx or www.cdc.gov/nchs/data/nhanes/nhanes_05_06/vid_d.pdf (18). According to the Endocrine Society Clinical Practice Guidelines, vitamin D status was categorized into three groups: deficiency (<49.9 nmol/L; to convert from nmol/L to ng/mL, divided by 2.5), insufficient (50.0–74.9 nmol/L), and sufficient (≥75.0 nmol/L) (13).

### 2.6 Covariates

Potential confounders were evaluated as covariates. Questionnaires collected information on age, sex, race (Non-Hispanic White, Non-Hispanic Black, Mexican-American, other Hispanic, or other race), educational level (less than high school, high school or equivalent, college or above), family income-to-poverty ratio (PIR) (<1.3, 1.3–3.5, or >3.5), smoking status (never smoker, former smoker, or current smoker), and leisure-time physical activity (inactive, moderately active, or vigorously active). Drinking status was categorized into four groups following prior NHANES-based studies (19): never (fewer than 12 drinks in a lifetime), former (no drinking in the past year but ≥12 drinks previously), light (up to 1 drink/day for women or 2 for men), and heavy (more than 1 drink/day for women or 2 for men). Laboratory analysis covariates included serum total cholesterol (TC), triglycerides (TG), serum uric acid (UA), and glycosylated hemoglobin (HbA1c). Hypertension was defined based on previous NHANES research (20) as any of the following: self-reported physician diagnosis, current use of antihypertensive medication, systolic blood pressure ≥140 mmHg, or diastolic blood pressure ≥90 mmHg. DM status was defined according to prior NHANES research (21) as either a self-reported physician diagnosis or current use of insulin or oral hypoglycemic agents. CVD was defined based on self-reported history of coronary heart disease, angina, myocardial infarction, or stroke, following previous studies using NHANES data (22). The participants were asked, “Has a doctor or other health professional ever told you that you have congestive heart failure (CHF)/coronary heart disease (CHD)/angina/heart attack/stroke?” Participants who answered “yes” to any of the above questions were considered patients with CVD. Daily dietary variables assessed included dietary vitamin D intake, total energy, protein, carbohydrates, total sugars, and total fat.

### 2.7 Statistical analyses

As the microbial intake variable was derived from the first 24-h dietary recall only, we used the corresponding Day 1 dietary weights (WTDRD1), in accordance with NHANES analytic guidelines. Continuous variables are presented as the mean (standard deviation) or median (interquartile range), while categorical variables are presented as a number (percentages).

To facilitate the analysis, the dietary intake of MedHi underwent log_10_-transformation after adding 0.1 g and was then analyzed as a continuous variable. For log-transformed MedHi intake, regression coefficients represent the absolute change in the outcome (e.g., BMI and WC) associated with each 1-unit increase in log₁₀ (MedHi + offset), which corresponds to a 10-fold increase in dietary live microbe intake. These regression models were adjusted for covariates as outlined in Tables 1–4. For regression models that measure grams MedHi consumed as a continuous exposure, regression coefficients are reported as one-unit increment in log_10_-transformation. These coefficients indicate the adjusted mean difference in the outcome for a one-unit increment in the log_10_-transformation of exposure. In regression models using the three-level classification of patients based on dietary intakes, non-consumers (G1) serve as the reference group. Regression coefficients are reported for the two indicator variables: one for participants with intakes above zero but below the median (G2) and another for participants with intakes at or above the median (G3), both included simultaneously in the model.

**Table 1:** Regression analysis showing the association between log10-transformed MedHi (g) and BMI, WC.

Variables	Per one-unit increment in log10-transformed MedHi							
	Model 1		Model 2		Model 3		Model 4	
	*β* (95% CI)	*p*	*β* (95% CI)	*p*	*β* (95% CI)	*p*	*β* (95% CI)	*p*
BMI	−0.26 [−0.34, −0.17]	<0.001	−0.21 [−0.29, −0.13]	<0.001	−0.17 [−0.25, −0.10]	<0.001	−0.15 [−0.22, −0.08]	<0.001
WC	−0.69 [−0.90, −0.47]	<0.001	−0.56 [−0.75, −0.37]	<0.001	−0.48 [−0.65, −0.32]	<0.001	−0.42 [−0.58, −0.26]	<0.001

Model 1: Crude model. Model 2: Adjusted for age, sex, race, education, smoking status, drinking status, and PIR. Model 3: Adjusted for Model 2 plus energy intake (kcal/day), CHOL, TG, HbA1c, uric acid, diabetes, hypertension, CVD, and months of blood collection. Model 4: Adjusted for Model 3 plus serum vitamin D.

**Table 2:** Regression analysis showing the association between dietary live microbe intake and obesity, and abdominal obesity.

Variables	Model 1		Model 2		Model 3		Model 4	
	OR (95% CI)	*p*	OR (95% CI)	*p*	OR (95% CI)	*p*	OR (95% CI)	*p*
Obesity								
Log10 transformed MedHi (g)								
	0.91 [0.89, 0.94]	<0.001	0.93 [0.90, 0.96]	<0.001	0.93 [0.90, 0.96]	<0.001	0.94 [0.91, 0.97]	<0.001
Categories								
G1	1		1		1		1	
G2	0.95 [0.83, 1.09]	0.497	0.99 [0.86, 1.14]	0.862	1.01 [0.87, 1.16]	0.941	1.01 [0.87, 1.18]	0.849
G3	0.75 [0.68, 0.83]	<0.001	0.80 [0.72, 0.88]	<0.001	0.81 [0.73, 0.89]	<0.001	0.82 [0.74, 0.91]	<0.001
*p* for trend	<0.001		<0.001		<0.001		<0.001	
Dietary live microbe intake group								
Low	1		1		1		1	
Moderate	0.83 [0.75, 0.91]	<0.001	0.85 [0.77, 0.95]	0.003	0.86 [0.77, 0.95]	0.005	0.88 [0.79, 0.98]	0.018
High	0.74 [0.67, 0.81]	<0.001	0.81 [0.73, 0.89]	<0.001	0.83 [0.74, 0.92]	0.001	0.84 [0.75, 0.94]	0.002
*p* for trend	<0.001		<0.001		<0.001		<0.001	
Abdominal obesity								
Log10 transformed MedHi (g)								
	0.96 [0.93, 0.98]	0.003	0.92 [0.90, 0.95]	<0.001	0.93 [0.90, 0.96]	<0.001	0.94 [0.91, 0.97]	<0.001
Categories								
G1	1		1		1		1	
G2	1.02 [0.91, 1.14]	0.733	0.99 [0.87, 1.12]	0.851	1.00 [0.88, 1.14]	0.993	1.01 [0.88, 1.16]	0.904
G3	0.88 [0.81, 0.96]	0.005	0.79 [0.72, 0.87]	<0.001	0.81 [0.73, 0.89]	<0.001	0.83 [0.75, 0.91]	<0.001
*p* for trend	0.004		<0.001		<0.001		<0.001	
Dietary live microbe intake group								
Low	1		1		1		1	
Moderate	0.93 [0.84, 1.02]	0.13	0.83 [0.75, 0.92]	0.001	0.84 [0.75, 0.93]	0.001	0.86 [0.77, 0.96]	0.006
High	0.88 [0.79, 0.97]	0.01	0.83 [0.74, 0.92]	0.001	0.85 [0.76, 0.96]	0.01	0.87 [0.77, 0.98]	0.021
*p* for trend	0.009		<0.001		0.007		0.017	

Model 1: Crude model. Model 2: Adjusted for age, sex, race, education, smoking status, drinking status, PIR, and recreational activity. Model 3: Adjusted for Model 2 plus energy intake (kcal/day), CHOL, TG, HbA1c, uric acid, diabetes, hypertension, CVD, and months of blood collection. Model 4: Adjusted for Model 3 plus serum vitamin D.

**Table 3:** Regression analysis showing the association between serum vitamin D (nmol/L) and obesity.

Variables	Model 1		Model 2		Model 3		Model 4	
	*β* (95% CI)	*p*	*β* (95% CI)	*p*	*β* (95% CI)	*p*	*β* (95% CI)	*p*
Per ten-unit increment in serum vitamin D (nmol/L)								
BMI	−0.41 [−0.47, −0.36]	<0.001	−0.43 [−0.49, −0.37]	<0.001	−0.34 [−0.40, −0.29]	<0.001	−0.34 [−0.39, −0.28]	<0.001
WC	−0.78 [−0.92, −0.65]	<0.001	−1.06 [−1.19, −0.92]	<0.001	−0.84 [−0.97, −0.72]	<0.001	−0.83 [−0.95, −0.70]	<0.001

Variables	Model 1		Model 2		Model 3		Model 4	
	OR (95% CI)	*p*	OR (95% CI)	*p*	OR (95% CI)	*p*	OR (95% CI)	*p*
Obesity								
Per ten-unit increment in serum vitamin D (nmol/L)								
	0.87 [0.85, 0.89]	<0.001	0.87 [0.85, 0.89]	<0.001	0.88 [0.86, 0.90]	<0.001	0.88 [0.86, 0.90]	<0.001
Serum vitamin D group (nmol/L)								
<50	1		1		1		1	
50–74.9	0.66 [0.59, 0.73]	<0.001	0.68 [0.61, 0.77]	<0.001	0.73 [0.64, 0.82]	<0.001	0.73 [0.65, 0.82]	<0.001
≥75	0.44 [0.40, 0.49]	<0.001	0.44 [0.39, 0.50]	<0.001	0.48 [0.42, 0.54]	<0.001	0.48 [0.42, 0.55]	<0.001
*p* for trend	<0.001		<0.001		<0.001		<0.001	
Abdominal obesity								
Per ten-unit increment in serum vitamin D (nmol/L)								
	0.93 [0.92, 0.95]	<0.001	0.87 [0.85, 0.89]	<0.001	0.88 [0.86, 0.90]	<0.001	0.88 [0.86, 0.90]	<0.001
Serum vitamin group D (nmol/L)								
<50	1		1		1		1	
50–74.9	0.73 [0.66, 0.80]	<0.001	0.70 [0.63, 0.78]	<0.001	0.73 [0.65, 0.82]	<0.001	0.73 [0.65, 0.83]	<0.001
≥75	0.65 [0.58, 0.72]	<0.001	0.46 [0.40, 0.52]	<0.001	0.50 [0.43, 0.58]	<0.001	0.50 [0.43, 0.59]	<0.001
*p* for trend	<0.001		<0.001		<0.001		<0.001	

Model 1: Crude model. Model 2: Adjusted for age, sex, race, education, smoking status, drinking status, PIR, and recreational activity. Model 3: Adjusted for Model 2 plus energy intake (kcal/day), CHOL, TG, HbA1c, uric acid, diabetes, hypertension, CVD, and months of blood collection. Model 4: Adjusted for Model 3 plus dietary live microbe intake.

**Table 4:** Regression analysis showing the association between log10-transformed MedHi (g) and serum vitamin D.

Variables	Per one-unit increment in log10-transformed MedHi							
	Model 1		Model 2		Model 3		Model 4	
	*β* (95% CI)	*p*	*β* (95% CI)	*p*	*β* (95% CI)	*p*	*β* (95% CI)	*p*
Serum vitamin D (nmol/L)	2.34 [2.01, 2.68]	<0.001	0.90 [0.58, 1.23]	<0.001	0.77 [0.45, 1.09]	<0.001	0.70 [0.38, 1.02]	<0.001

Model 1: Crude model. Model 2: Adjusted for age, sex, race, education, smoking status, drinking status, PIR, and recreational activity. Model 3: Adjusted for Model 2 plus energy intake (kcal/day), CHOL, TG, HbA1c, uric acid, diabetes, hypertension, CVD, and months of blood collection. Model 4: Adjusted for Model 3 plus dietary vitamin D intake.

Generalized linear regression models and restricted cubic spline (RCS) models were employed to evaluate the associations between obesity and dietary intake of MedHi, obesity and serum vitamin D levels, and the relationship between serum vitamin D levels and dietary intake of MedHi.

To account for NHANES’s complex sampling design, Day 1 dietary weights (WTDRD1) were applied during model estimation. Consistent with previous NHANES-based mediation studies, weights were incorporated at the estimation stage rather than during resampling, ensuring population-level interpretability. Covariates were selected *a priori* based on previous NHANES-based studies, biological plausibility, and their known associations with dietary intake, vitamin D metabolism, and obesity outcomes. This analysis was conducted using the mediation package in R software (version 4.5.0) to estimate the indirect, direct, and total effects (23). Both the mediator and outcome models were specified using linear regression (svylm()), and bootstrapped confidence intervals were computed based on 1,000 simulations. In the mediation model, the total effect (c) of dietary live microbe intake on obesity was decomposed into a direct effect (c′) not mediated by serum vitamin D, and an indirect effect (a × b) mediated through serum vitamin D. Here, “a” represents the association between live microbe intake and serum vitamin D, and “b” represents the association between serum vitamin D and obesity, adjusting for the exposure. The same set of covariates was consistently adjusted in each path (a-path, b-path, c-path, and c′-path), including: age, sex, race, education, smoking status, drinking status, PIR and recreational activity, energy intake (kcal/day), CHOL, TG, HbA1c, uric acid, diabetes, hypertension, CVD, and months of blood collection and dietary vitamin D intake. The mediated proportion was calculated as the ratio of the indirect effect to the sum of the indirect and direct effects, multiplied by 100%. Two-sided *p*-values less than 0.05 were considered statistically significant.

We hypothesized that serum vitamin D levels mediate the association between dietary live microbe intake and obesity based on previous evidence, suggesting that both live microbial intake and vitamin D are associated with adiposity, and that gut microbiota may influence vitamin D absorption or metabolism.

### 2.8 Assessment of multicollinearity

To address potential multicollinearity among covariates, especially among dietary variables, we calculated variance inflation factors (VIFs) in the regression models. As shown in Supplementary Table S5, several dietary intake variables—including protein, carbohydrate, total sugars, total fat, and dietary fiber—exhibited high VIF values (e.g., VIF for energy intake = 55.54; carbohydrate = 36.96), indicating substantial collinearity. To mitigate this issue and avoid model overfitting, we included only total energy intake (kcal/day) in the final multivariable models, which showed acceptable collinearity (VIF = 1.57). This approach ensured model stability while retaining relevant nutritional adjustment.

## 3 Results

### 3.1 Participant characteristics

The demographic characteristics of 18,099 participants from the NHANES, surveyed between 2007 and 2018, are detailed in Table 5. The average age was 47.64 ± 0.27 years, with males comprising 54.79% of the cohort. A majority, 95.74%, had received education at a high school level or higher. The racial composition was predominantly White (69.76%) and Black (10.09%), with 79.85% identifying as non-Hispanic. Mexican Americans represented 7.91%, other Hispanics 5.31%, and other racial groups 6.93%. Additionally, 44.57% of participants had a history of tobacco use and 77.31% reported alcohol consumption. Prevalence rates for DM and CVD were 13.70 and 8.33%, respectively, while hypertension was reported by 35.73%. The average BMI was 28.64 ± 0.08 kg/m^2^, with no significant difference between men (28.63 ± 0.09) and women (28.65 ± 0.11; *p* = 0.85). In contrast, the mean waist circumference (WC) differed significantly by sex, measuring 101.51 ± 0.25 cm in men and 96.13 ± 0.26 cm in women (*p* < 0.001). Detailed sex-specific anthropometric data are presented in Supplementary Table S6. Across live microbe intake groups, race (SMD = 0.306), education (0.277), and income-to-poverty ratio (0.278) showed notable differences. High intake was more common among non-Hispanic White, and among those with higher education and income. Smaller imbalances were seen in sex, physical activity, and serum vitamin D levels. Overall, most SMDs were <0.2, indicating minimal baseline differences between groups.

**Table 5:** The clinical characteristics of participants stratified by dietary live microbe intake groups.

Variables	Total	Live microbe intake			*p*	SMD
		Low	Moderate	High		
Age (years)	47.64 (0.27)	45.87 (0.35)	49.18 (0.34)	47.63 (0.41)	<0.0001	0.094
Sex (*N*, %)					<0.0001	0.116
Male	9,024 (49.56)	3,655 (54.79)	3,551 (47.95)	1,818 (45.37)		
Female	9,075 (50.44)	3,135 (45.21)	3,732 (52.05)	2,208 (54.63)		
Race (*N*, %)					<0.0001	0.306
Non-Hispanic White	8,056 (69.76)	2,678 (63.69)	3,196 (69.10)	2,182 (78.24)		
Non-Hispanic Black	3,653 (10.09)	1,884 (15.30)	1,257 (8.78)	512 (5.50)		
Mexican-American	2,629 (7.91)	874 (7.87)	1,279 (9.64)	476 (5.49)		
Other Hispanic	1,815 (5.31)	645 (5.63)	770 (5.53)	400 (4.59)		
Other race	1,946 (6.93)	709 (7.51)	781 (6.96)	456 (6.18)		
Education (*N*, %)					<0.0001	0.277
Less than high school	1,545 (4.26)	652 (5.39)	709 (4.88)	184 (1.99)		
High school or equivalent	6,585 (33.00)	2,888 (41.30)	2,547 (31.15)	1,150 (25.37)		
College or above	9,969 (62.73)	3,250 (53.31)	4,027 (63.97)	2,692 (72.64)		
PIR (*N*, %)					<0.0001	0.278
<1.3	5,534 (20.35)	2,526 (27.38)	2,073 (18.40)	935 (14.43)		
1.3–3.5	6,891 (35.82)	2,657 (38.57)	2,816 (35.82)	1,418 (32.42)		
>3.5	5,674 (43.82)	1,607 (34.05)	2,394 (45.78)	1,673 (53.15)		
Smoking status (*N*, %)					<0.0001	0.176
Never	9,976 (55.43)	3,470 (50.35)	4,140 (57.10)	2,366 (59.35)		
Former	4,465 (25.36)	1,530 (22.95)	1,920 (27.16)	1,015 (25.77)		
Current	3,658 (19.21)	1,790 (26.70)	1,223 (15.74)	645 (14.88)		
Drinking status (*N*, %)					<0.0001	0.16
Never alcohol intake	2,412 (10.18)	949 (11.13)	1,019 (10.88)	444 (8.00)		
Former alcohol intake	2,783 (12.51)	1,195 (14.34)	1,104 (12.65)	484 (10.03)		
Mild alcohol intake	6,447 (38.64)	2,144 (33.48)	2,671 (39.64)	1,632 (43.61)		
Heavy alcohol intake	6,457 (38.67)	2,502 (41.06)	2,489 (36.82)	1,466 (38.36)		
Recreational activity (*N*, %)					<0.0001	0.193
No	9,139 (44.49)	3,870 (52.87)	3,545 (42.43)	1,724 (37.04)		
Moderate	4,925 (29.45)	1,639 (26.16)	2,067 (30.24)	1,219 (32.40)		
Vigorous	4,035 (26.06)	1,281 (20.97)	1,671 (27.33)	1,083 (30.56)		
Diabetes (*N*, %)					<0.0001	0.085
No	14,788 (86.30)	5,534 (86.38)	5,835 (84.71)	3,419 (88.46)		
Yes	3,311 (13.70)	1,256 (13.62)	1,448 (15.29)	607 (11.54)		
Hypertension (*N*, %)					0.17	0.059
No	10,762 (64.27)	3,969 (63.66)	4,267 (63.68)	2,526 (65.85)		
Yes	7,337 (35.73)	2,821 (36.34)	3,016 (36.32)	1,500 (34.15)		
CVD					<0.001	0.081
No	16,181 (91.67)	5,989 (90.88)	6,494 (91.10)	3,698 (93.46)		
Yes	1,918 (8.33)	801 (9.12)	789 (8.90)	328 (6.54)		
Obesity (*N*, %)					<0.0001	0.082
No	11,239 (62.81)	4,039 (59.11)	4,567 (63.66)	2,633 (66.18)		
Yes	6,860 (37.19)	2,751 (40.89)	2,716 (36.34)	1,393 (33.82)		
Months of blood collection (*N*, %)					0.001	0.068
May–October	9,426 (56.20)	3,345 (52.57)	3,894 (57.74)	2,187 (58.52)		
November–April	8,673 (43.80)	3,445 (47.43)	3,389 (42.26)	1,839 (41.48)		
BMI (kg/m^2^)	28.64 (0.08)	29.09 (0.11)	28.49 (0.10)	28.30 (0.13)	<0.0001	0.072
WC (cm)	98.80 (0.21)	100.03 (0.30)	98.40 (0.26)	97.85 (0.34)	<0.0001	0.084
TC (mmol/L)	5.03 (0.01)	4.97 (0.02)	5.03 (0.02)	5.09 (0.02)	0.002	0.052
TG (mmol/L)	1.74 (0.02)	1.78 (0.02)	1.75 (0.03)	1.68 (0.03)	0.02	0.024
UA (μmol/L)	5.43 (0.02)	5.54 (0.03)	5.40 (0.03)	5.34 (0.03)	<0.0001	0.099
HbA1C (%)	5.61 (0.01)	5.64 (0.01)	5.65 (0.01)	5.54 (0.02)	<0.0001	0.094
Dietary vitamin D intake (mcg)	4.58 (0.04)	4.27 (0.07)	4.68 (0.07)	4.82 (0.10)	<0.0001	0.085
Serum vitamin D (nmol/L)	64.94 (0.53)	60.73 (0.65)	66.59 (0.59)	67.80 (0.57)	<0.0001	0.195
Energy (kcal/day)	2098.05 (8.99)	2063.07 (15.37)	2091.90 (11.54)	2150.20 (16.08)	<0.001	0.102
MedHi (g/day)	0.98 (0.02)	0.00 (0.00)	1.34 (0.02)	1.68 (0.03)	<0.0001	1.334
Protein (g/day)	81.84 (0.36)	78.14 (0.52)	82.09 (0.58)	86.07 (0.69)	<0.0001	0.157
Carbohydrate (g/day)	248.86 (1.11)	250.00 (2.01)	249.11 (1.44)	247.11 (2.01)	0.52	0.029
Total sugars (g/day)	108.52 (0.72)	111.42 (1.35)	106.59 (0.91)	107.67 (1.21)	0.01	0.025
Total fat (g/day)	81.12 (0.43)	78.13 (0.68)	80.93 (0.58)	85.11 (0.74)	<0.0001	0.144

PIR, poverty income ratio; CVD, cardiovascular disease; BMI, body mass index; WC, waist circumference; TC, total cholesterol; TG, triglyceride; UA, uric acid. Data are presented as means (SE) for continuous measures and numbers (percentages) for categorical measures.

To further explore group differences by obesity status, baseline characteristics are presented in Supplementary Table S1.

### 3.2 Association between dietary intake of live microbes with obesity

**Figure 3:**
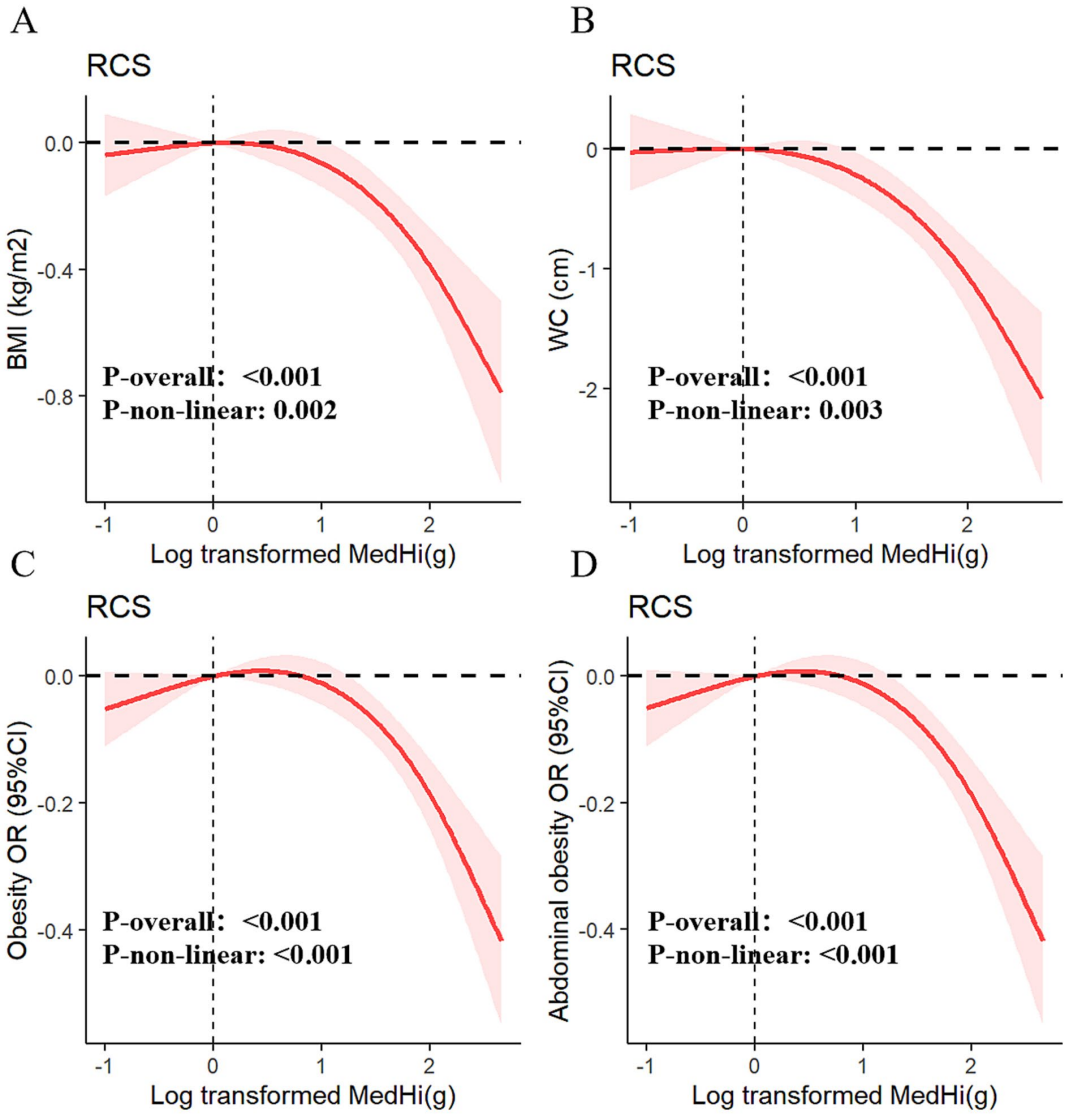
Dose–response relationships of MedHi intake with BMI **(A)**, WC **(B)**, obesity **(C)**, and abdominal obesity **(D)**.

Multivariable generalized linear regression analysis reveals a negative association between MedHi consumption and body metrics. We used a series of progressively adjusted models to examine the robustness of these associations: Model 1: Crude model; Model 2: Adjusted for age, sex, race, education, smoking status, drinking status, income, and recreational activity; Model 3: Adjusted for Model 2 plus energy intake (kcal/day), CHOL, TG, HbA1c, uric acid, diabetes, hypertension, CVD, and months of blood collection; Model 4: Adjusted for Model 3 plus serum vitamin D. In the fully adjusted model (Model 4), each one-unit increment in log_10_-transformed MedHi corresponds to a decrease of 0.15 kg/m^2^ in BMI (95% CI: −0.22, −0.08) and a reduction of 0.42 cm in WC (95% CI: −0.58, −0.26), as shown in Table 1. Furthermore, adjusted RCS models revealed a nonlinear association between MedHi consumption and BMI, with significant nonlinearity observed (Figure 3A), while the relationship between MedHi consumption and WC appeared linear (Figure 3B).

Figures 3C,D demonstrate a dose–response relationship between MedHi intake and both obesity and abdominal obesity. After multivariate adjustment, a nonlinear association was observed between MedHi intake and obesity, and a similar situation occurred with MedHi and abdominal obesity. Each one-unit increment in the log_10_-transformed MedHi was associated with a 6% reduced risk of both obesity and abdominal obesity, as detailed in Table 2.

Additionally, when MedHi consumption was categorized, the risk of both obesity and abdominal obesity was significantly lower in the G3 group (highest consumption category) compared to the G1 group (non-consumers) across all four models. The analysis revealed that the risks of both obesity and abdominal obesity were significantly lower in the moderate and high intake groups compared with the low intake group.

### 3.3 Association between serum vitamin D with obesity

**Figure 4:**
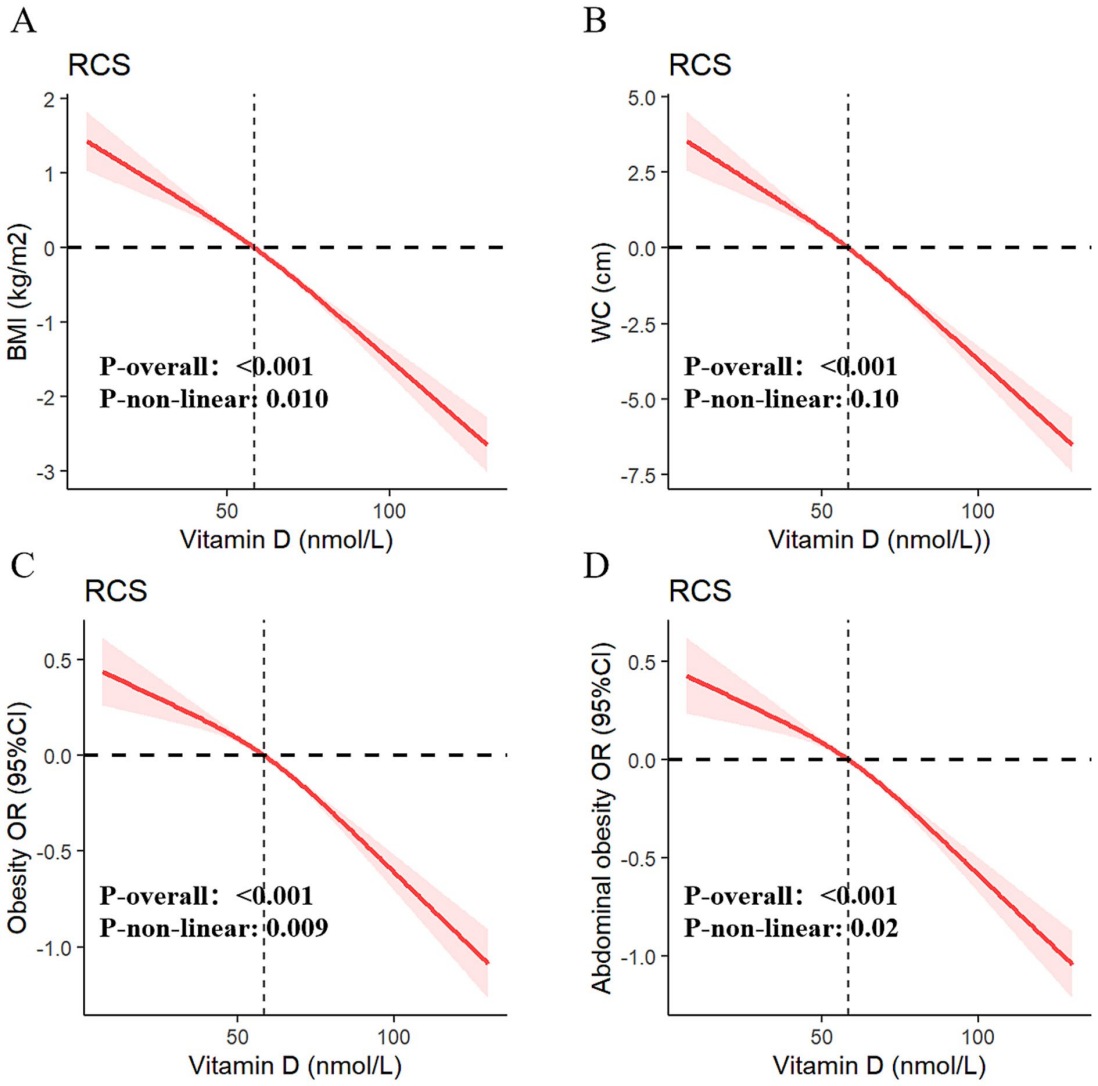
Dose–response relationships of serum vitamin D with BMI **(A)**, WC **(B)**, obesity **(C)**, and abdominal obesity **(D)**.

Table 3 presents the results of multivariable generalized linear regression analyses assessing the associations between serum vitamin D levels and obesity-related indicators. In the fully adjusted model, each 10 nmol/L increase in serum vitamin D was significantly associated with a decrease in BMI (*β* = −0.34, *p* < 0.001) and WC (*β* = −0.83, *p* < 0.001), indicating a strong inverse relationship. This linear association was further supported by adjusted RCS models (Figures 4A,B).

Additionally, univariate and multivariable weighted logistic regression analyses showed that higher serum vitamin D levels were significantly associated with lower odds of both general obesity and abdominal obesity. The RCS curves (Figures 4C,D) confirmed robust negative dose–response relationships between vitamin D levels and both outcomes. When serum vitamin D was categorized into deficiency, insufficiency, and sufficiency groups, participants in the higher-level categories consistently showed lower risks of obesity and abdominal obesity across all four models. Full model specifications and estimates are provided in Table 3.

### 3.4 Association between dietary intake of live microbes with serum vitamin D

**Figure 5:**
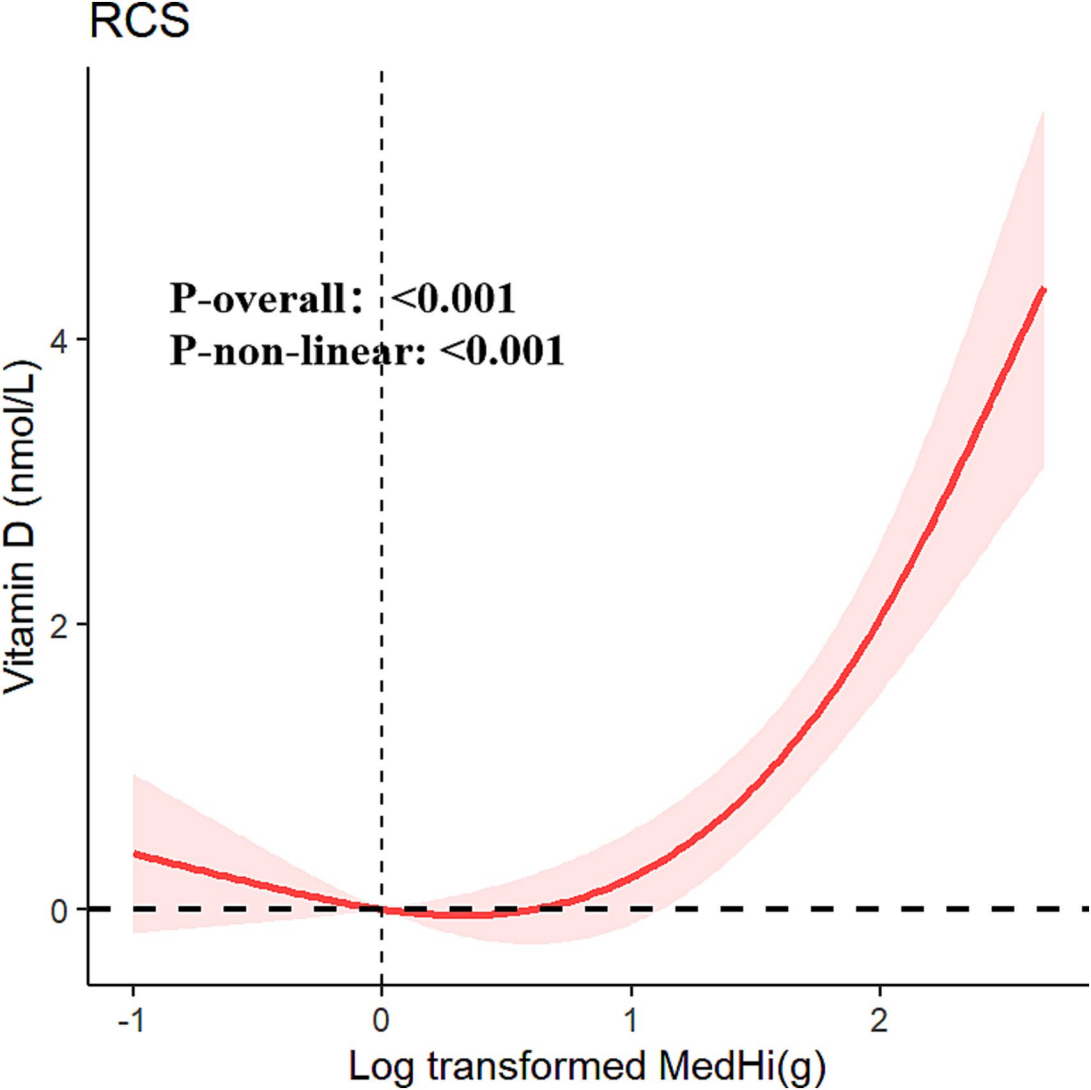
Restricted cubic spline fitting for the association between dietary live microbe intake with serum vitamin D adjusted for all covariables.

The multivariable generalized linear regression coefficients for serum vitamin D levels, corresponding to a one-unit increment in the log_10_-transformed MedHi, are detailed in Table 4. The results consistently show a positive association across all four models. Specifically, each one-unit increment in the log_10_-transformed MedHi consumption was associated with increases in serum vitamin D levels of 2.34 nmol/L (95% CI: 2.01, 2.68), 0.90 nmol/L (95% CI: 0.58, 1.23), 0.77 nmol/L (95% CI: 0.45, 1.09), and 0.70 nmol/L (95% CI: 0.38, 1.02), respectively. Further analysis using adjusted RCS models indicated that the relationship between MedHi consumption and serum vitamin D levels is nonlinear, with a significant test for nonlinearity as shown in Figure 5.

### 3.5 Mediation analysis

**Figure 6:**
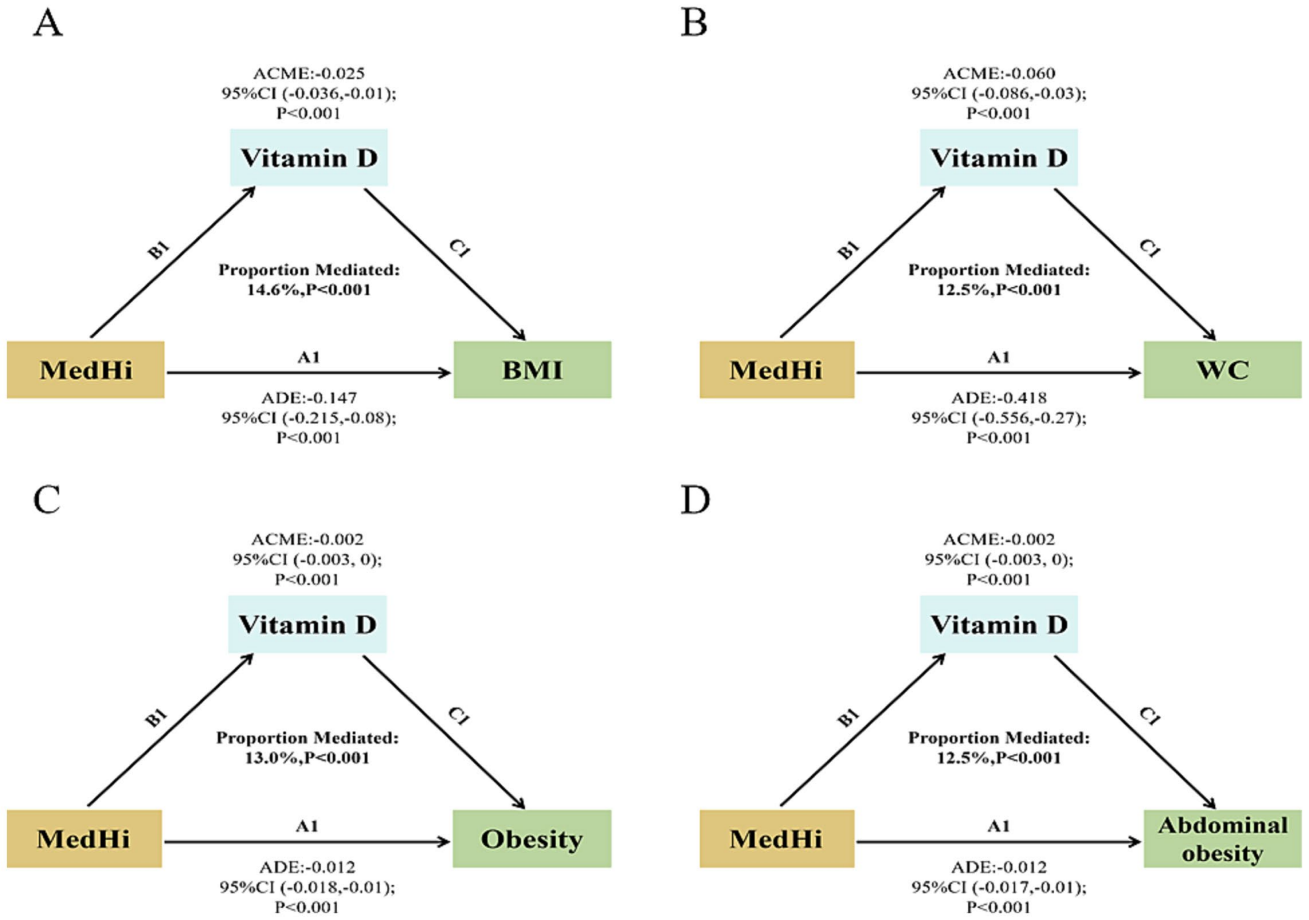
Path diagram of the mediation effect. **(A)** Serum vitamin D mediated effects on the associations of dietary live microbe intake with BMI. **(B)** Serum vitamin D mediated effects on the associations of dietary live microbe intake with WC. **(C)** Serum vitamin D mediated effects on the associations of dietary live microbe intake with obesity. **(D)** Serum vitamin D mediated effects on the associations of dietary live microbe intake with abdominal obesity.

Mediation analyses were conducted to assess the potential mediation effects of serum vitamin D on the relationships between dietary intake of live microbes and various obesity-related metrics. The analysis revealed significant mediation effects of serum vitamin D on the associations with BMI, WC, obesity, and abdominal obesity. The proportions of mediation were 14.6, 12.5, 13.0, and 12.5% respectively, all achieving statistical significance (*p* < 0.001), as shown in Figures 6A–D. To evaluate the role of vitamin D in the interaction between MedHi intake and obesity metrics, mediation models were thoroughly adjusted for a comprehensive set of variables. These included age, sex, race, education, smoking status, drinking status, PIR and recreational activity, energy intake (kcal/day), CHOL, TG, HbA1c, uric acid, diabetes, hypertension, CVD, and months of blood collection and dietary vitamin D intake.

### 3.6 Sensitivity analyses

To assess the robustness of our findings, we performed sensitivity analyses. First, we tested for effect modification by baseline vitamin D status (deficient vs. sufficient) by including an interaction term between MedHi intake and vitamin D status. No significant interaction was observed for obesity, indicating consistency across subgroups (Supplementary Figures S1, S2).

To test the robustness of the log-transformation applied to MedHi intake, we conducted sensitivity analyses using alternative offset values. Specifically, MedHi was log-transformed using three approaches: log₁₀ (MedHi + 0.01), log₁₀ (MedHi + 0.001), and ln (MedHi + 0.1). As shown in Supplementary Table S2, all models yielded consistent and statistically significant associations with both general and abdominal obesity, indicating that our findings are not sensitive to the specific offset used. Similarly, the results of the mediation analyses remained stable across these transformations (Supplementary Figures S4A–F).

Additionally, to further test the robustness of our findings, we conducted a sensitivity analysis excluding participants with implausible total energy intake values (<800 kcal/day or >4,200 kcal/day for men; <600 kcal/day or >3,500 kcal/day for women). The associations between dietary live microbe intake, serum vitamin D levels, and obesity remained consistent with the main analyses. Importantly, the mediation effect of serum vitamin D persisted after excluding these extreme values, as shown in Supplementary Table S3 and Supplementary Figures S4G,H. These results indicate that our findings are not driven by outliers or potential misreporting in dietary intake data.

As part of the sensitivity analyses, we explored whether our findings were robust under alternative obesity definitions. Specifically, we applied the Chinese diagnostic criteria for obesity (BMI ≥28 kg/m^2^) in place of the WHO cutoff (BMI ≥30 kg/m^2^). The results remained directionally consistent with the main analysis, indicating that the observed associations and mediation effects were not sensitive to the choice of obesity classification. Detailed findings are provided in Supplementary Table S4 and Supplementary Figure S3.

To examine the robustness of the mediation model, we tested for exposure-mediator interaction by including an interaction term between dietary live microbe intake and serum vitamin D levels. The interaction was not statistically significant (*p* for interaction = 0.89), suggesting that the effect of dietary live microbes on obesity does not differ across serum vitamin D levels. Detailed results are presented in Supplementary Figures S1, S2.

## 4 Discussion

NHANES provides extensive data critical for shaping nutrition and health policies. In this study, we integrated this data to conduct a comprehensive assessment of a large cohort. For the first time, our mediation analysis demonstrated that serum vitamin D levels can partially mediate the relationship between dietary intake of live microorganisms and obesity in a nationally representative sample of U.S. adults.

The gut microbiota is increasingly recognized as a crucial regulator of host health, akin to the role of vitamin D in linking various metabolic pathways. Probiotics, which modulate the gut microbiome, have been shown to influence host health and potentially reduce obesity risk (24). Consumption of yogurt has been associated with reductions in adiposity factors such as BMI and WC (25), as well as improvements in cardio-metabolic risk factors, including blood pressure, lipid profiles, and glucose levels (26). Importantly, foodborne microbes have been found to survive transit through the digestive system and remain metabolically active within the gut, illustrating how the gut microbiome can rapidly respond to dietary changes (27).

Specifically, each one-unit increase in log10-transformed MedHi intake was associated with a 0.15 kg/m^2^ reduction in BMI, which equates to approximately 0.43 kg of body weight for an individual 1.70 m in height—highlighting the potential for modest but meaningful public health impact at the population level. Dietary live microbes may influence obesity risk via modulation of gut microbiota and subsequent effects on vitamin D metabolism. Beneficial microbes such as *Lactobacillus* and *Bifidobacterium* can enhance intestinal barrier integrity and vitamin D receptor expression (28). They also produce short-chain fatty acids (SCFAs), which improve vitamin D absorption and activation (29). In addition, gut microbiota influence bile acid metabolism, further facilitating vitamin D uptake (30). Both SCFAs and vitamin D possess anti-inflammatory and anti-adipogenic properties (31). A bidirectional relationship may also exist, as vitamin D can shape gut microbiota through immunomodulatory effects (32). These interconnected pathways support the observed mediation effect.

The meta-analysis results (33, 34) support an inverse relationship between obesity measures and vitamin D levels. Historically, research has primarily focused on the intake of vitamin D and its role in obesity management, with less emphasis on enhancing serum vitamin D levels through alternative means. Our study clarifies that the intake of live microbes unidirectionally influences vitamin D levels and obesity, establishing a potential causal relationship between live microbe intake and these factors. We found that the relationship between live microbe intake and obesity is mediated by serum vitamin D levels, highlighting mediating factors that elucidate the biological pathways connecting live microbe intake to obesity outcomes. Although we adjusted for several dietary and lifestyle factors, it is possible that the observed associations may partially reflect overall diet quality rather than the specific effects of live microbes. Individuals with high live microbe intake often consume diets richer in fiber, fruits, and fermented foods, which are independently associated with improved vitamin D status and reduced obesity risk. Future studies should incorporate formal diet quality indices to help disentangle these overlapping dietary influences. Furthermore, although many vitamin D supplementation trials (35–37) successfully raised vitamin D concentrations, they often failed to yield the anticipated health benefits. This discrepancy suggests that a diet rich in live microbes might be more effective in reducing obesity incidence than relying solely on vitamin D supplementation.

Importantly, the observed mediation proportions by serum vitamin D in our study (12.5–14.6%) fall within the range reported by other nutritional epidemiology studies using NHANES data. For example, Lin et al. (38) found that BMI mediated approximately 21.3% of the total effect of sugar-sweetened beverage intake on decreased serum 25(OH)D levels in U.S. adults. Similarly, Yang et al. (39) reported that BMI partially mediated the relationship between sodium intake and systolic blood pressure, contributing a substantial proportion of the total effect (based on NHANES 2007–2016 data). These comparisons suggest that mediation proportions in the 10–20% range are plausible and meaningful in complex dietary pathways, supporting the role of vitamin D as a modest yet biologically relevant mediator in the link between live microbe intake and obesity.

Our study has several limitations. First, its cross-sectional design limits causal inference. Although we hypothesized a directional pathway—from dietary live microbe intake to serum vitamin D levels and then to obesity—based on prior biological and longitudinal evidence, the temporal sequence cannot be firmly established using NHANES data. Reverse causality remains possible, though we attempted to mitigate this through sensitivity analyses and flexible modeling. Prospective studies are needed to validate these associations. Second, the diversity of geographic regions, dietary patterns, and lifestyle behaviors may jointly influence the mediating variable (serum vitamin D), thereby affecting multiple interrelated factors in our model. Third, although our microbial exposure classification was based on a validated methodology, it did not differentiate among specific types of live microbes. Certain strains—particularly those with probiotic properties—may exert more pronounced metabolic or anti-inflammatory effects than others. Future research incorporating strain-level microbial identification and functional analysis is needed to clarify these differential effects. Third, the diversity of geographic regions, diets, and lifestyles might synergistically influence the “mediation variable” in our analysis, affecting multiple interrelated factors. Fourth, another potential explanation for our findings is the presence of co-occurring nutrients in live microbe-rich foods. For instance, yogurt and other fermented dairy products not only contain probiotics but are also rich in calcium, protein, and bioactive peptides, which may independently impact adiposity and vitamin D metabolism. Similarly, fermented vegetables and soy-based foods contain fiber, vitamins, and phytonutrients that may act as confounders. Although our models adjusted for total energy intake, residual confounding by specific co-nutrients cannot be fully excluded. Fifth, differences in vitamin D metabolism across racial and ethnic groups may influence the observed associations. For example, non-Hispanic Black individuals typically exhibit lower circulating 25(OH)D concentrations without corresponding increases in adverse health outcomes—likely due to differences in vitamin D-binding protein levels and tissue-level sensitivity to vitamin D (40). Such physiological variations may influence vitamin D bioavailability and its biological activity, potentially modifying its mediating role. While our analyses adjusted for race/ethnicity, stratified mediation analyses were not performed due to limited statistical power in certain subgroups. Future studies with larger and more diverse populations are warranted to explore potential effect modification by racial/ethnic group. Finally, longitudinal studies are essential to clarify the long-term interplay between live microbe intake, vitamin D status, and obesity outcomes.

## 5 Conclusion

This study revealed that the negative correlation between dietary intake of live microbes and obesity is mediated by vitamin D. Foods with higher microbial concentrations could be beneficial in reducing the incidence of obesity.

## Data Availability

The datasets presented in this study can be found in online repositories. The names of the repository/repositories and accession number(s) can be found in the article/Supplementary material.

## Footnotes

^1^ http://www.cdc.gov/nchs/nhanes/

## Supplementary material

SUPPLEMENTARY FIGURE S1 Association between dietary live microbe intake with obesity, stratified by age, gender, race, vitamin D status and months of blood collection.

SUPPLEMENTARY FIGURE S2 Association between dietary live microbe intake with abdominal obesity, stratified by age, gender, race, vitamin D status and months of blood collection.

SUPPLEMENTARY FIGURE S3 Path diagram of the mediation effect. Serum vitamin D mediated effects on the associations of dietary live microbe intake with obesity (Chinese obesity diagnostic criteria: BMI ≥28 kg/m²).

SUPPLEMENTARY FIGURE S4 Path diagram of the mediation effect. **(A,B)** Dietary intake of MedHi underwent log10-transformation after adding 0.01 g. **(C,D)** Dietary intake of MedHi underwent log10-transformation after adding 0.001 g. **(E,F)** Dietary intake of MedHi underwent natural log-transformation after adding 0.1 g. **(G,H)** Excluded participants with implausible energy intake.
