# Supplementary figures and images for: Exploring the mediating role of serum vitamin D in the link between dietary live microbes intake and obesity: a cross-sectional real-world study
Source: Front Nutr. 2025 Aug 29;12:1588700. doi: 10.3389/fnut.2025.1588700 (PMC12425993; doi:10.3389/fnut.2025.1588700)

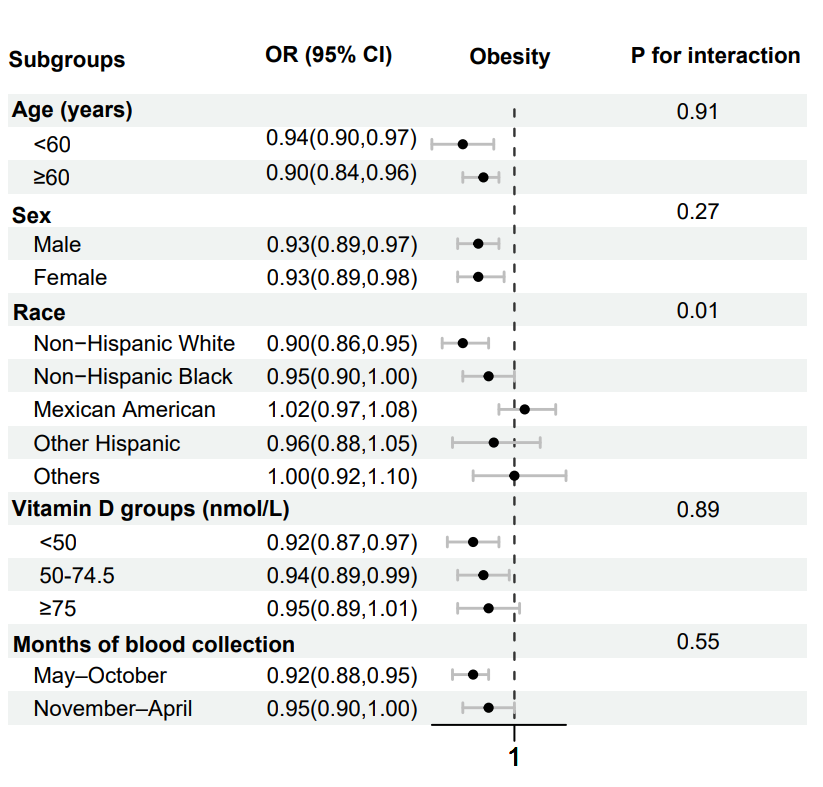

Supplement: SUPPLEMENTARY FIGURE S1 — Association between dietary live microbe intake with obesity, stratified by age, gender, race, vitamin D status and months of blood collection. [file Image_1.TIF]

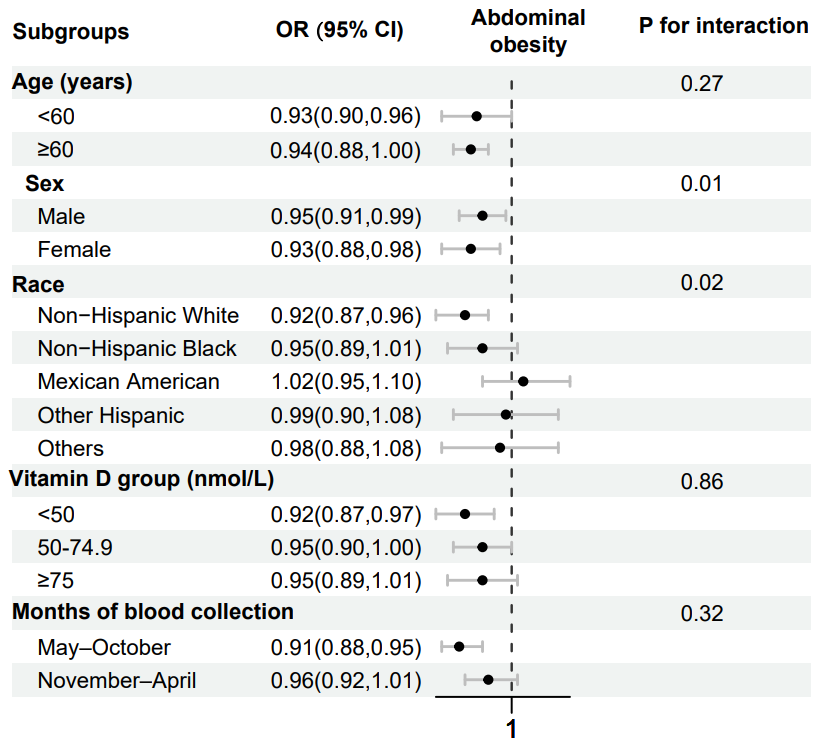

Supplement: SUPPLEMENTARY FIGURE S2 — Association between dietary live microbe intake with abdominal obesity, stratified by age, gender, race, vitamin D status and months of blood collection. [file Image_2.TIF]

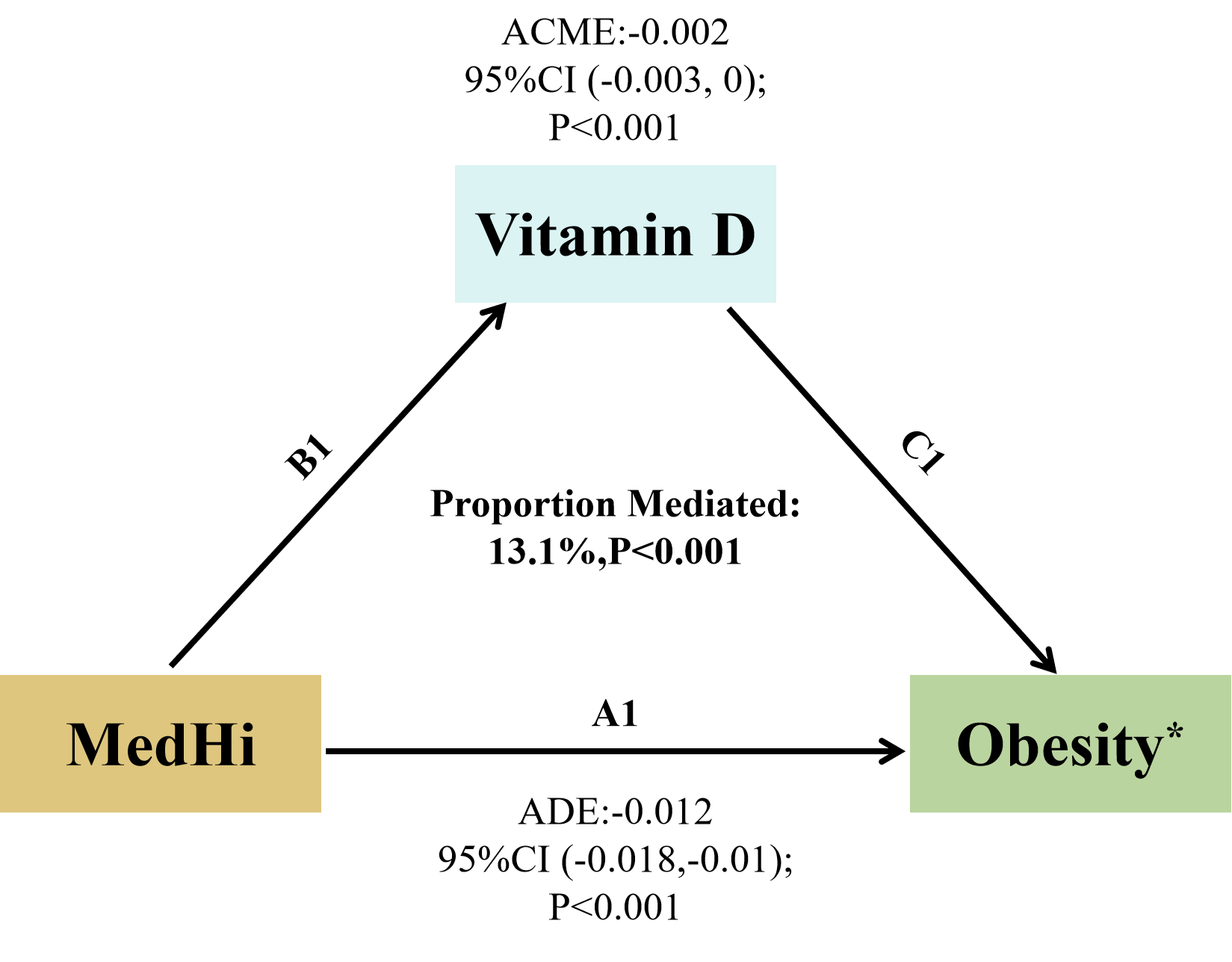

Supplement: SUPPLEMENTARY FIGURE S3 — Path diagram of the mediation effect. Serum vitamin D mediated effects on the associations of dietary live microbe intake with obesity (Chinese obesity diagnostic criteria: BMI ≥28 kg/m²). [file Image_3.TIF]

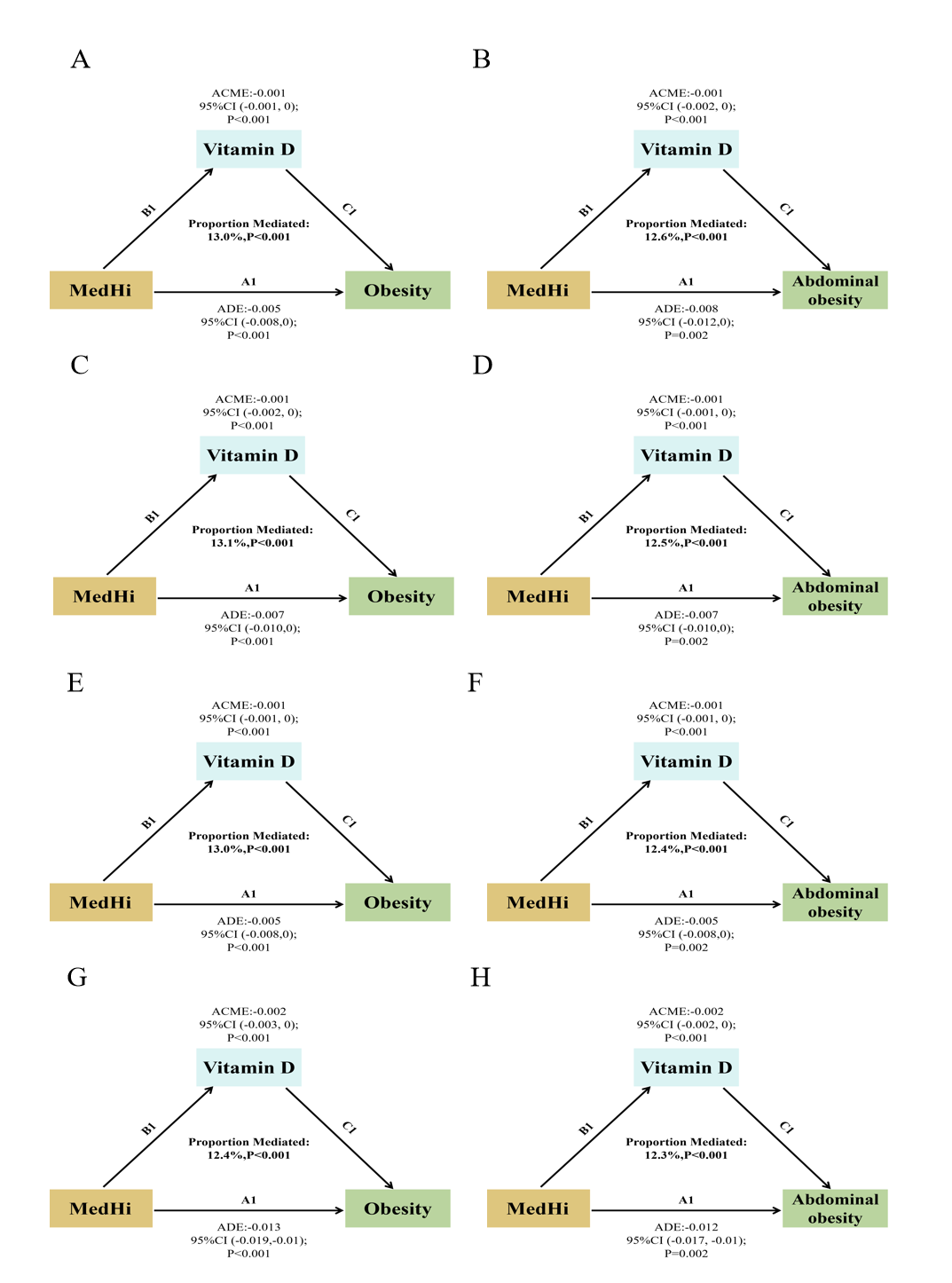

Supplement: SUPPLEMENTARY FIGURE S4 — Path diagram of the mediation effect. (A,B) Dietary intake of MedHi underwent log10-transformation after adding 0.01 g. (C,D) Dietary intake of MedHi underwent log10-transformation after adding 0.001 g. (E,F) Dietary intake of MedHi underwent natural log-transformation after adding 0.1 g. (G,H) Excluded participants with implausible energy intake. [file Image_4.TIF]
