# Supplementary material for: Exploring the mediating role of serum vitamin D in the link between dietary live microbes intake and obesity: a cross-sectional real-world study
Source: Front Nutr. 2025 Aug 29;12:1588700. doi: 10.3389/fnut.2025.1588700 (PMC12425993; doi:10.3389/fnut.2025.1588700)
Supplement: Supplementary file 5 [file Table_1.DOCX]

Table S1 The clinical characteristics of participants stratified by obesity status.

| Variables | Total | Non-obesity | Obesity | P |
| --- | --- | --- | --- | --- |
| Age (years) | 47.64(0.27) | 46.92(0.32) | 48.85(0.31) | < 0.0001 |
| Sex (N, %) |  |  |  | 0.09 |
| Male | 9024(49.56) | 5871(50.27) | 3153(48.35) |  |
| Female | 9075(50.44) | 5368(49.73) | 3707(51.65) |  |
| Race (N, %) |  |  |  | < 0.0001 |
| Non-Hispanic White | 8056(69.76) | 5134(70.90) | 2922(67.84) |  |
| Non-Hispanic Black | 3653(10.09) | 2000( 8.73) | 1653(12.38) |  |
| Mexican-American | 2629( 7.91) | 1464(6.96) | 1165(9.52) |  |
| Other Hispanic | 1815( 5.31) | 1098(5.15) | 717(5.57) |  |
| Other Race | 1946( 6.93) | 1543(8.26) | 403(4.68) |  |
| Education (N, %) |  |  |  | < 0.0001 |
| Less than high school | 1545( 4.26) | 922(4.12) | 623(4.50) |  |
| High school or equivalent | 6585(33.00) | 3946(31.19) | 2639(36.07) |  |
| College or above | 9969(62.73) | 6371(64.69) | 3598(59.43) |  |
| PIR (N, %) |  |  |  | < 0.0001 |
| <1.3 | 5534(20.35) | 3380(19.96) | 2154(21.01) |  |
| 1.3-3.5 | 6891(35.82) | 4177(34.47) | 2714(38.12) |  |
| >3.5 | 5674(43.82) | 3682(45.57) | 1992(40.87) |  |
| Smoking status (N, %) |  |  |  | < 0.0001 |
| Never | 9976(55.43) | 6175(55.68) | 3801(55.01) |  |
| Former | 4465(25.36) | 2626(23.91) | 1839(27.81) |  |
| Current | 3658(19.21) | 2438(20.42) | 1220(17.18) |  |
| Drinking status (N, %) |  |  |  | < 0.0001 |
| Never | 2412(10.18) | 1469( 9.93) | 943(10.60) |  |
| Former | 2783(12.51) | 1551(11.01) | 1232(15.03) |  |
| Mild | 6447(38.64) | 4191(40.02) | 2256(36.31) |  |
| Heavy | 6457(38.67) | 4028(39.04) | 2429(38.06) |  |
| Recreational activity (N, %) |  |  |  | < 0.0001 |
| No | 9139(44.49) | 5236(39.56) | 3903(52.81) |  |
| Moderate | 4925(29.45) | 3099(29.62) | 1826(29.15) |  |
| Vigorous | 4035(26.06) | 2904(30.81) | 1131(18.04) |  |
| Diabetes (N, %) |  |  |  | < 0.0001 |
| No | 14788(86.30) | 9808(91.63) | 4980(77.29) |  |
| Yes | 3311(13.70) | 1431( 8.37) | 1880(22.71) |  |
| Hypertension (N, %) |  |  |  | < 0.0001 |
| No | 10762(64.27) | 7433(71.25) | 3329(52.46) |  |
| Yes | 7337(35.73) | 3806(28.75) | 3531(47.54) |  |
| CVD |  |  |  | < 0.0001 |
| No | 16181(91.67) | 10189(93.05) | 5992(89.34) |  |
| Yes | 1918( 8.33) | 1050( 6.95) | 868(10.66) |  |
| Months of blood collection (N, %) |  |  |  | 0.45 |
| May–October | 9426(56.20) | 5908(56.60) | 3518(55.53) |  |
| November–April | 8673(43.80) | 5331(43.40) | 3342(44.47) |  |
| Dietary live microbe intake |  |  |  | < 0.0001 |
| Low | 6790(33.83) | 4039(31.84) | 2751(37.20) |  |
| Moderate | 7283(38.88) | 4567(39.40) | 2716(37.99) |  |
| High | 4026(27.29) | 2633(28.76) | 1393(24.81) |  |
| BMI (kg/m2) | 28.64(0.08) | 25.03(0.05) | 34.75(0.06) | < 0.0001 |
| WC (cm) | 98.80(0.21) | 90.42(0.17) | 112.95(0.17) | < 0.0001 |
| TC (mmol/L) | 5.03(0.01) | 5.02(0.02) | 5.04(0.02) | 0.4 |
| TG (mmol/L) | 1.74(0.02) | 1.54(0.02) | 2.08(0.03) | < 0.0001 |
| UA (umol/L) | 5.43(0.02) | 5.20(0.02) | 5.83(0.02) | < 0.0001 |
| HbA1C (%) | 5.61(0.01) | 5.48(0.01) | 5.84(0.02) | < 0.0001 |
| dietary vitamin D intake (mcg) | 4.58(0.04) | 4.75(0.06) | 4.30(0.07) | < 0.0001 |
| Serum vitamin D(nmol/L) | 64.94(0.53) | 67.93(0.56) | 59.88(0.62) | < 0.0001 |
| Energy (kcal/d) | 2098.05(8.99) | 2108.72(11.47) | 2080.04(13.79) | 0.11 |
| Protein (g/d) | 81.84(0.36) | 81.76(0.47) | 81.98(0.53) | 0.75 |
| Carbohydrate (g/d) | 248.86(1.11) | 252.07(1.38) | 243.46(1.82) | < 0.001 |
| Total sugars (g/d) | 108.52(0.72) | 109.97(0.84) | 106.08(1.08) | 0.002 |
| Total fat (g/d) | 81.12(0.43) | 80.20(0.52) | 82.68(0.68) | 0.003 |

Data are presented as means (SE) for continuous measures and numbers(percentage) for categorical measures.

Abbreviations: PIR: poverty income ratio，CVD: cardiovascular disease, BMI: body mass index, WC: waist circumference, TC: total cholesterol, TG: triglyceride, UA: uric acid.

Table S2 Association between dietary live microbe intake and obesity.

|  | **Obesity** | | **Abdominal obesity** | |
| --- | --- | --- | --- | --- |
| Variables | OR (95%CI) | *P* | OR (95%CI) | *P* |
| MedHi ^a^ | 0.96 [0.93, 0.98] | <0.001 | 0.95 [0.93, 0.98] | <0.001 |
| MedHi ^b^ | 0.97 [0.95, 0.98] | <0.001 | 0.96 [0.95, 0.98] | <0.001 |
| MedHi ^c^ | 0.97 [0.96, 0.99] | <0.001 | 0.97 [0.96, 0.99] | <0.001 |

a: Dietary intake of MedHi underwent log10-transformation after adding 0.01g;

b: Dietary intake of MedHi underwent log10-transformation after adding 0.001g;

c: Dietary intake of MedHi underwent natural log-transformation after adding 0.1g;

Adjusted for age, sex, race, education, smoking status, drinking status, PIR and recreational activity, energy intake (kcal/d), CHOL, TG, HbA1c, uric acid, diabetes, hypertension, CVD and months of blood collection and serum vitamin D.

Table S3 Association between dietary live microbe intake and obesity, abdominal obesity excluded participants with implausible energy intake.

|  | **Obesity** | | **Abdominal obesity** | |
| --- | --- | --- | --- | --- |
|  | OR (95%CI) | *P* | OR (95%CI) | *P* |
| **Continues** | 0.94 [0.91, 0.97] | <0.001 | 0.94 [0.91, 0.97] | <0.001 |
| **Categories** |  |  |  |  |
| G1 | 1 |  | 1 |  |
| G2 | 1.01 [0.86, 1.18] | 0.92 | 1.01 [0.87, 1.16] | 0.937 |
| G3 | 0.82 [0.73, 0.91] | <0.001 | 0.83 [0.75, 0.91] | <0.001 |
| p for trend | <0.001 |  | <0.001 |  |
| **Dietary Live Microbe Intake group** | | |  |  |
| Low | 1 |  | 1 |  |
| Moderate | 0.87 [0.78, 0.97] | 0.011 | 0.85 [0.76, 0.95] | 0.004 |
| High | 0.83 [0.74, 0.94] | 0.003 | 0.88 [0.78, 0.99] | 0.032 |
| p for trend | <0.001 |  | <0.001 |  |

Adjusted for age, sex, race, education, smoking status, drinking status, PIR and recreational activity, energy intake (kcal/d), CHOL, TG, HbA1c, uric acid, diabetes, hypertension, CVD and months of blood collection and serum vitamin D.

Table S4 Association between dietary live microbe intake and obesity (Chinese obesity diagnostic criteria: BMI ≥28 kg/m²).

| Variables | Obesity | |
| --- | --- | --- |
|  | OR (95%CI) | P |
| Continues | 0.94 [0.91, 0.97] | <0.001 |
| Categories |  |  |
| G1 | 1 |  |
| G2 | 1.03 [0.91, 1.18] | 0.62 |
| G3 | 0.83 [0.75, 0.92] | 0.001 |
| p for trend | <0.001 |  |
| Dietary Live Microbe Intake group | | |
| Low | 1 |  |
| Moderate | 0.86 [0.78, 0.95] | 0.005 |
| High | 0.88 [0.79, 0.99] | 0.04 |
| p for trend | <0.001 |  |

Adjusted for age, sex, race, education, smoking status, drinking status, PIR and recreational activity, energy intake (kcal/d), CHOL, TG, HbA1c, uric acid, diabetes, hypertension, CVD and months of blood collection and serum vitamin D.

Table S5 VIF values for the regression analysis of dietary live microbe intake and obesity.

| Variables | VIF ^a^ | VIF ^b^ |
| --- | --- | --- |
| Serum vitamin D | 2.526364 | 2.06623 |
| Age | 2.80858 | 2.696377 |
| Sex |  |  |
| Male | 1 | 1 |
| Female | 2.264665 | 2.201383 |
| eth |  |  |
| Non-Hispanic White | 1 |  |
| Non-Hispanic Black | 2.532616 | 2.175516 |
| Mexican American | 2.957996 | 2.615475 |
| Other Hispanic | 2.587107 | 2.145513 |
| Others | 2.048476 | 1.783934 |
| Education |  |  |
| Less than high school | 1 | 1 |
| High school or equivalent | 6.394239 | 4.98312 |
| College or above | 5.934612 | 5.133615 |
| Family income-to-poverty ratio |  |  |
| <1.3 | 1 |  |
| 1.3-3.5 | 2.216336 | 2.094404 |
| >3.5 | 2.543547 | 2.390177 |
| Smoking status |  |  |
| Never | 1 | 1 |
| Former | 2.012396 | 1.69887 |
| Current | 3.281311 | 2.229899 |
| Drinking status |  |  |
| Never | 1 | 1 |
| Former | 2.029057 | 1.95161 |
| Mild | 4.169931 | 3.752701 |
| Heavy | 5.426633 | 4.493504 |
| Recreational activity |  |  |
| No | 1 | 1 |
| Moderate | 1.927953 | 1.81175 |
| Vigorous | 2.179345 | 2.07291 |
| Diabetes (Yes) | 3.117525 | 3.257774 |
| Hypertension (Yes) | 1.740035 | 1.612566 |
| CVD (Yes) | 2.006334 | 1.772656 |
| Months of blood collection | 2.596466 | 2.192191 |
| TC (mmol/L) | 1.50156 | 1.454608 |
| TG (mmol/L) | 2.204913 | 1.767836 |
| UA (umol/L) | 2.398457 | 1.975336 |
| HbA1C (%) | 3.176054 | 3.098139 |
| Energy (kcal/d) | 55.540598 | 1.566999 |
| Protein (g/d) | 7.840549 |  |
| Carbohydrate (g/d) | 36.956797 |  |
| Total sugars (g/d) | 10.565431 |  |
| Total fat (g/d) | 11.776012 |  |
| Dietary fiber (g/d) | 5.895211 |  |

a : Adjusted for age, sex, race, education, smoking status, drinking status, income and recreational activity, CHOL, TG, HbA1c, uric acid, diabetes, hypertension, CVD and months of blood collection, serum vitamin D, energy intake (kcal/d), protein (g/d) , carbohydrate (g/d) , total sugars (g/d) , total fat (g/d), dietary fiber (g/d).

b : Adjusted for age, sex, race, education, smoking status, drinking status, income and recreational activity, CHOL, TG, HbA1c, uric acid, diabetes, hypertension, CVD and months of blood collection, serum vitamin D, energy intake (kcal/d).

Table S6 BMI and WC stratified by sex.

| **Variables** | **Total** | **Female** | **Male** | ***P*** |
| --- | --- | --- | --- | --- |
| BMI | 28.64(0.08) | 28.65(0.11) | 28.63(0.09) | 0.85 |
| WC | 98.80(0.21) | 96.13(0.26) | 101.51(0.25) | < 0.0001 |
